# Formalin as a Surgical Dressing1Read at the Thirty-sixth Annual Meeting of the American Veterinary Medical Association, New York City, September, 1899.

**Published:** 1899-09

**Authors:** Charles H. Higgins

**Affiliations:** Pathologist to the Department of Agriculture, Dominion of Canada


### FORMALIN AS A SURGICAL DRESSING.[1]

By Charles H. Higgins, B.S., D.V.S.,
PATHOLOGIST TO THE DEPARTMENT OF AGRICULTURE, DOMINION OF CANADA.

Formalin, a derivative of wood-alcohol, has for the past few years been used to a considerable extent as a preservative for tissues in pathological laboratories the world over. It has the power of preserving tissues, retaining them at more nearly their normal color than any other general preservative. In hospitals it is used in solution for the sterilization of instruments, while the gas is used to disinfect wards, clothing, etc. It is beginning to be used extensively also by various health boards and sanitary commissions.

As a strict surgical dressing we see little or no mention of this agent.

There are constantly occurring in the practice of every veterinarian wounds of a more or less serious nature, caused by an animal coming in contact with some foreign object (as in runaways and other accidents), which it is necessary to treat as suppurating wounds, owing to their size and the impossibility in many cases of

[1] Read at the Thirty-sixth Annual Meeting of the American Veterinary Medical Association, New York City, September, 1899.

obtaining a perfectly aseptic condition. There are also fistulæ of various portions of the body which yield very slowly to the best treatment.

During the past spring a case came to my notice, one afternoon, which was interesting and yielded very readily to a formalin treatment after other remedies had failed to give good results.

A mare, about eight years old, was tied to a fence while the owner went into an adjoining field in order to look at some cattle which he proposed buying. On returning to the place where he had left his horse he found the animal down, with the right shaft of the buggy entering the neck a distance of about eight inches. He loosened the harness, and the animal got up without assistance. He again hitched her into the buggy and drove to my hospital, a distance of about four miles. When I first saw the animal it seemed that the treatment would be a very simple matter, as the wound did not seem very extensive, there being an opening in the skin about an inch and a half in diameter, extending forward and upward about three inches. The wound was cleansed with a creolin solution and packed with iodoform gauze. This dressing was allowed to remain a day and a half, and upon its removal the cavity had extended downward, forming a small pocket containing pus. An incision was made to insure drainage, and dressed in the same manner as before. The animal was very stiff on the fore-legs, owing to the fact that the wound involved the "levator humeri" muscle.

From this time on the wound was dressed every day. On the fifth day after the animal was taken into the hospital I was obliged to leave town for a period of three days, which necessitated leaving the animal in the hands of a faithful, though inexperienced person. Upon my return, which was the eighth day from the occurrence of the accident, I found the animal in a very bad condition, the cavity having extended downward between the muscles, beneath the vertebræ to the other side of the neck, where a very slight pressure would force the pus out of the opening. It was necessary to do something, and that immediately, or the animal would be worthless. An incision downward from the first wound, laying open about eight inches of the neck in the region of the fifth cervical vertebra, almost severing the levator humeri muscle. Upon an exploration of the wound it was found that the transverse process of the fifth cervical vertebra was broken completely off. This was removed and the wound thoroughly syringed out with a 3 per cent. solution of formalin, nothing else being done at this time.

The next morning the wound was again dressed with a 1 per cent. solution of formalin, this treatment being continued up to the time that the animal was discharged, four days later, with instructions to use a solution (1 per cent. formalin), given daily.

After the first injection of a formalin solution there was a decided improvement in the condition of the animal and in the appearance of the wound. The suppuration ceased in quantity from day to day, and in two weeks from the time that the animal was discharged there was but a very small wound upon the original site, without pus-formation.

This is my first and only experience with this new material, and from the success in this one case I think that it deserves a place in the hospital of every veterinarian. The treatment is a very painful one to the patient, but the good results more than counterbalance the suffering entailed.

With formalin we get an astringent action of a very powerful antiseptic, with hardening of the exposed tissue, and do not get the caustic effect that is obtained by the use of silver nitrate and very strong solutions of bichloride of mercury. The antiseptic action, owing to the manner in which the tissues are hardened, lasts for a number of days; perhaps not from the direct action as an antiseptic, but in the resistance which the hardened tissues present to the infecting microbe.

