# Corrections

**Published:** 1899-11

**Authors:** 


### CORRECTIONS.

September, 1899, number, page 567, footnote, "Read at the Thirty-sixth Annual Meeting of the American Veterinary Medical Association, New York City, September, 1899," should not appear as such.

September, 1899, number, page 569, "(1 per cent. formalin) given daily" should read "given (1 per cent. formalin) daily."

---

Prof. R. S. Huidekoper will give a special course of lectures this winter on Anatomy at the United States College of Veterinary Surgeons, Washington, D. C.

